# The genetic basis of hyaline fibromatosis syndrome in patients from a consanguineous background: a case series

**DOI:** 10.1186/s12881-018-0581-1

**Published:** 2018-05-25

**Authors:** Leila Youssefian, Hassan Vahidnezhad, Andrew Touati, Vahid Ziaee, Amir Hossein Saeidian, Sara Pajouhanfar, Sirous Zeinali, Jouni Uitto

**Affiliations:** 1 Department of Dermatology and Cutaneous Biology, Sidney Kimmel Medical College, Thomas Jefferson University, 233 S. 10th Street, Suite 450 BLSB, Philadelphia, PA 19107 USA; 2 Biotechnology Research Center, Department of Molecular Medicine, Pasteur Institute of Iran, Tehran, Iran; 3 Drexel University College of Medicine, Philadelphia, PA USA; 4 Department of Medical Genetics, School of Medicine, Tehran University of Medical Sciences, Tehran, Iran; 5 Department of Pediatrics, Children’s Medical Center, Pediatrics Center of Excellence, Tehran, Iran; 6 Kawsar Human Genetics Research Center, Tehran, Iran

**Keywords:** Hyaline fibromatosis syndrome, Genodermatoses, Mutation analysis, HFS, *ANTXR2* gene

## Abstract

**Background:**

Hyaline fibromatosis syndrome (HFS) is a rare heritable multi-systemic disorder with significant dermatologic manifestations. It is caused by mutations in *ANTXR2*, which encodes a transmembrane receptor involved in collagen VI regulation in the extracellular matrix. Over 40 mutations in the *ANTXR2* gene have been associated with cases of HFS. Variable severity of the disorder in different patients has been proposed to be related to the specific mutations in these patients and their location within the gene.

**Case presentation:**

In this report, we describe four cases of HFS from consanguineous backgrounds. Genetic analysis identified a novel homozygous frameshift deletion c.969del (p.Ile323Metfs*14) in one case, the previously reported mutation c.134 T > C (p.Leu45Pro) in another case, and the recurrent homozygous frameshift mutation c.1073dup (p.Ala359Cysfs*13) in two cases. The epidemiology of this latter mutation is of particular interest, as it is a candidate for inhibition of nonsense-mediated mRNA decay. Haplotype analysis was performed to determine the origin of this mutation in this consanguineous cohort, which suggested that it may develop sporadically in different populations.

**Conclusions:**

This information provides insights on genotype-phenotype correlations, identifies a previously unreported mutation in *ANTXR2,* and improves the understanding of a recurrent mutation in HFS.

## Background

Hyaline fibromatosis syndrome (HFS, OMIM# 228600) is a rare heritable disorder with variable severity and frequent lethality, characterized by thickened skin with nodules, papules and plaques, often with a periorificial and perianal distribution, gingival hypertrophy, and joint contractures. Osteopenia, predisposition to respiratory infections, and diarrhea are often present.

HFS is an autosomal recessive disorder, caused by mutations in the *ANTXR2* gene, also referred to as *CMG2*. This gene encodes ANTXR2, a type I transmembrane protein initially characterized for its role in angiogenesis and as a receptor for the anthrax toxin [1, 2]. While over 40 mutations in the *ANTXR2* gene have been reported in association with HFS so far, most of them (~70%) being missense and frameshift mutations scattered along the protein, the molecular pathogenesis of this disease has only recently begun to be understood. Specifically, ANTXR2 has been shown to act as a receptor for collagen VI, promoting lysosome-mediated degradation of collagen VI in the extracellular matrix [3], consistent with the finding that patients with HFS develop an accumulation of collagen VI [4]. Thus, defective collagen VI degradation due to nonfunctional ANTXR2 likely leads to collagen accumulation in patients’ tissues, resulting in the clinical manifestations of HFS.

In this report, we examine four patients from consanguineous Iranian backgrounds diagnosed with HFS, and present their clinical and genetic findings (Table 1).

**Table 1:** Clinical and genetic findings in four patients with HFS

	Case 1	Case 2	Case 3	Case 4
Mutation in the *ANTXR2* gene	c.969del (p.Ile323Metfs*14)	c.134 T > C (p.Leu45Pro)	c.1073dup (p.Ala359Cysfs*13)	c.1073dup (p.Ala359Cysfs*13)
Parental consanguinity	First cousin	First cousin	First cousin, once removed	First cousin
Status	Deceased (6 months)	Deceased (10 months)	Alive (2 years old)	Deceased (1.5 years)
Age of onset	3 months	1 month	Birth	Birth
Skin findings	Erythematous plaques on torso, hyperpigmentation, skin thickening	Perianal plaques, hyperpigmentation, skin thickening	Papular lesions on forehead, ears, and around the nose, hyperpigmentation, skin thickening	Perianal plaques, perioral papules, hyperpigmentation, skin thickening
Joint contractures/pain	+	+	+	+
Low bone density	+	–	–	–
Gingival hypertrophy	+	+	+	+
Recurrent diarrhea	+	+	–	+
Respiratory infections	+	+	+	+

## Case presentation

**Fig. 1:**
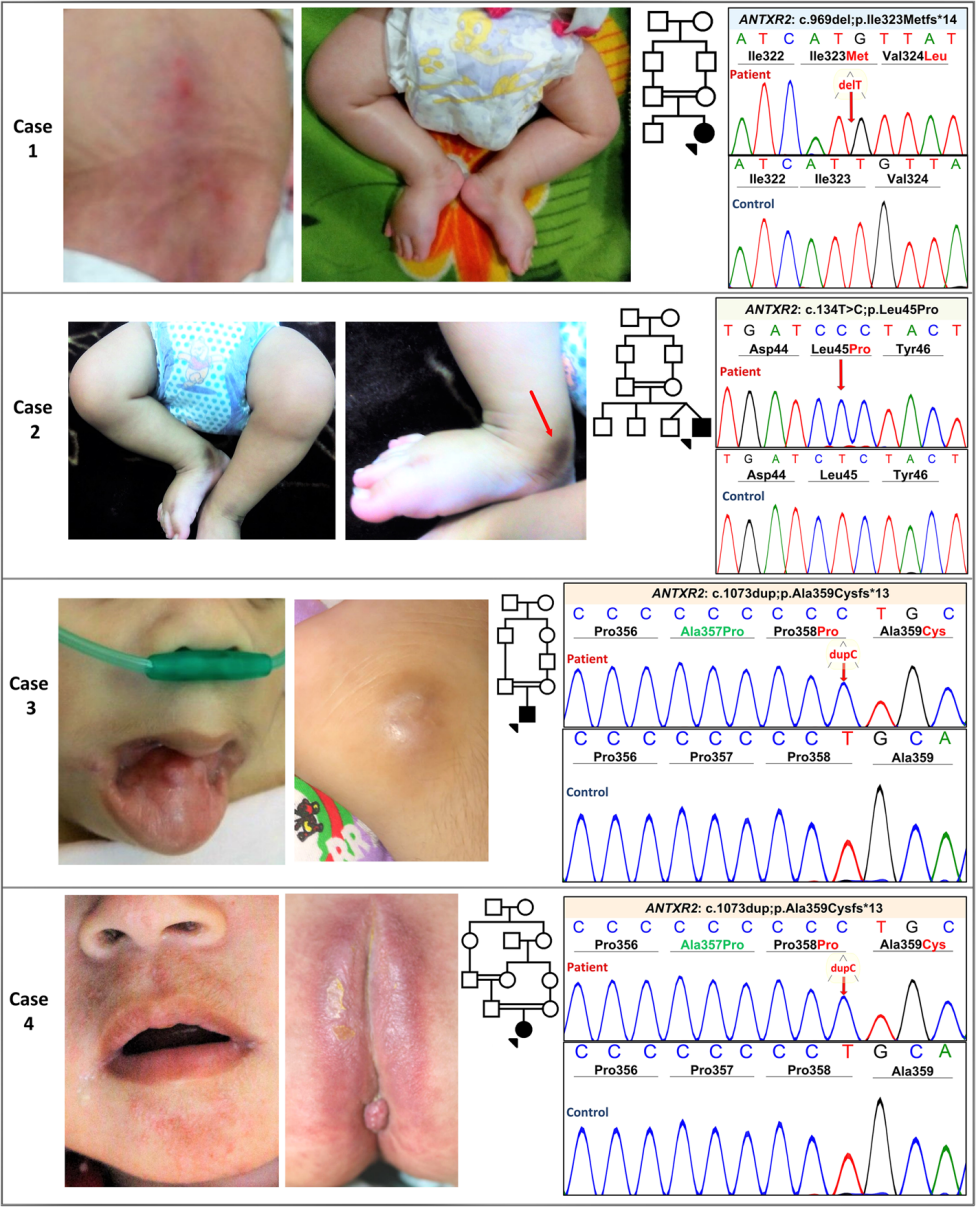
Clinical and genetic findings in four cases of HFS. Pedigrees displaying consanguinity, clinical findings, and Sanger sequencing of the identified *ANTXR2* mutations. In each case, the upper sequence panel represents the mutant allele, as compared to reference sequence in a healthy control below. **a** Clinical features of Case 1 included erythematous plaques, hyperpigmentation, and joint contractures of the lower extremities. Sanger sequencing identified a novel homozygous mutation, c.969del, which is predicted to result in truncated protein product, p.Ile323Metfs*14. **b** First cousin consanguinity, lower extremity contractures with hyperpigmentation over the medial malleolus, and Sanger sequencing of the c.134C > T (p.Leu45Pro) mutation in Case 2. **c** Case 3 developed flesh-colored papules on the face, including periauricular lesions, as well as gingival hyperplasia. Sequencing revealed the recurrent mutation c.1073dup (p.Ala359Cysfs*13). **d** Case 4 presented with characteristic perianal lesions and perioral papules. The same mutation as in Case 3 was identified by Sanger sequencing. The green in Case 3 and 4 represents the amino acid change, p.Ala357Pro, resulting from a common benign polymorphism, c.1069G > C (rs12647691)

Case 1 presented in infancy with dusky, erythematous plaques and hyperpigmentation over the torso and joints (Fig. 1a). Painful joint contractures, recurrent diarrhea, gingival hypertrophy, and recalcitrant upper respiratory tract infections were present. The patient died from infectious complications at 6 months of age.

Case 2 presented at 1 month of age with joint contractures, skin thickening, hyperpigmentation, and perianal plaques (Fig. 1b). The patient died at 10 months of age following recurrent respiratory infections and severe diarrhea.

Case 3 presented neonatally with painful contractures of the lower extremities (Fig. 1c). The patient developed papular lesions on the forehead, nose and ears, as well as hyperpigmentation over the medial malleoli. Gingival hyperplasia was present, and the patient developed frequent respiratory infections.

Case 4 presented in infancy with painful contractures of the legs and interphalangeal joints, perianal erythematous plaques, and perioral papules (Fig. 1d). Additional features included hyperpigmentation over the medial malleoli, gingival hypertrophy, recurrent diarrhea, and respiratory infections. The patient died at 18 months of age from infectious complications. On the basis of clinical presentations, HFS was suspected in all four cases.

Genetic analysis of DNA isolated from peripheral blood of each proband was performed using PCR-based amplification using 17 pairs of primers spanning all exons and ~ 50 bp of flanking intronic sequences of the *ANTXR2* gene (NM_058172; primer sequences available upon request). PCR products were bidirectionally sequenced using an automated sequencer (3730; Applied Biosystems, Foster City, CA, USA). Sequencing revealed a previously unreported homozygous frameshift deletion mutation c.969del (p.Ile323Metfs*14) in exon 12 in Case 1, and the previously reported homozygous missense mutation c.134 T > C (p.Leu45Pro) in exon 1 in Case 2 [5]. A homozygous frameshift insertion c.1073dup (p.Ala359Cysfs*13) in exon 13 was identified in Cases 3 and 4. This mutation has been identified in several previous cases of HFS, including an Iranian case reported by our laboratory [6]. The mutation occurs in a cytosine/guanosine rich region of DNA that has been suggested to be prone to mutational events including insertions and deletions [7, 8].

To determine whether this c.1073dup (p.Ala359Cysfs*13) mutation was due to independent mutational events at this hotspot, or due to a single distant founder effect mutation in the Iranian population, haplotype analysis was performed using a series of informative single nucleotide polymorphisms (SNPs) around the *ANTXR2* gene (Table 2). HapMap data (https://snpinfo.niehs.nih.gov/snpinfo/snptag.html) were used to choose tag SNPs, spanning a region of 2.91 Mb encompassing the *ANXTR2* gene. Selected tag SNPs were rs4692955, rs1493177, rs12509909, rs10011562, rs7685006, rs4975133, rs4975132, and rs4975131 (https://www.ncbi.nlm.nih.gov/SNP). Five intragenic SNPs in *ANTXR2* were additionally included as tag SNPs: rs13140055, rs4594664, rs11730210, rs4336166, and rs12647691 (https://www.ncbi.nlm.nih.gov/SNP). Overall, 13 SNPs were genotyped by PCR (primer sequences available upon request) and bidirectionally sequenced using an automated sequencer (3730; Applied Biosystems).

**Table 2:**
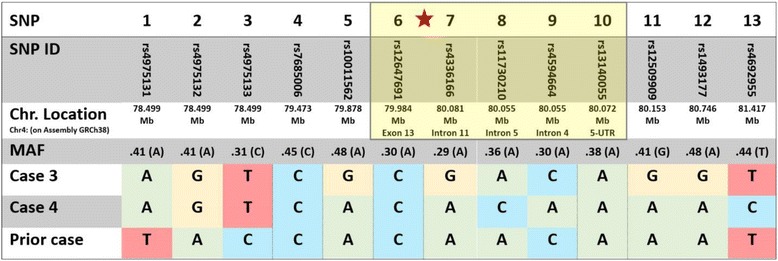
Haplotype analysis in cases with the c.1073dup mutation in *ANTXR2* gene

Haplotype analysis was performed in three cases of HFS homozygous for the c.1073dup (p.Ala359Cysfs*13) mutation in *ANTXR2*, including Case 3 and Case 4 from the present report, and a previously reported patient of Iranian descent with the identical mutation. Thirteen informative single nucleotide polymorphisms (SNPs) with minor allele frequencies (MAF) ranging from 0.29 to 0.48 in a 2.91 Mb region of chromosome 4 (https://www.ncbi.nlm.nih.gov/SNP) were sequenced in each case, including five SNPs within the *ANTXR2* gene (nos. 6-10, yellow highlight). The position of the c.1073dup mutation is indicated by a red asterisk. Analysis revealed that while each patient was homozygous for the allele of each included SNP as expected in consanguineous families, the SNP haplotype differed between these three cases. These data suggest an independent origin of this mutation event rather than being inherited from a common ancestor

Haplotype analysis was performed in Cases 3 and 4, as well as in our previously reported Iranian case of HFS with the same c.1073dup (p.Ala359Cysfs*13) mutation [6] (Table 2). Comparison of the haplotypes of these three Iranian patients showed a lack of haplotype conservation for SNPs in this region.

## Discussion and conclusions

Collectively, these cases add to the genetic understanding of HFS. Cases with mutations in the exons encoding the cytoplasmic tail of ANTXR2 protein have been reported to be clinically less severe than those with mutations upstream in the gene, which is likely to be due to the role of the cytoplasmic tail in receptor turnover, rather than directly in ligand-binding [9]. While Cases 3 and 4 harbored truncating mutations affecting the cytoplasmic tail, the upstream frameshift mutation in Case 1 is predicted to result in a truncated protein, likely impairing receptor stability in the cellular membrane. Case 2 harbored a mutation in the von Willebrand domain, essential for ligand binding, including to collagen VI [3]. Case 1 and 2 had the most severe phenotypes and earliest lethality, supporting the notion that mutations upstream of the cytoplasmic tail domain result in increased phenotypic severity.

The consequences of specific frameshift mutations in the *ANTXR2* gene have previously been examined, including the c.1073dup (p.Ala359Cysfs*13) mutation disclosed in Cases 3 and 4 [10]. Patients who are homozygous for this mutation have ANTXR2 mRNA expression levels that are half of those found in normal individuals [11]. However, the protein product of this mutated form of ANTXR2 has been shown to reach the cell membrane with partial functionality [10]. Thus, this mutation is a candidate for targeted inhibition of nonsense-mediated mRNA decay. The c.1073dup (p.Ala359Cysfs*13) mutation has been reported multiple times previously, and understanding the inheritance and epidemiology of this mutation in different populations is important in determining which HFS patients would benefit from potential therapy. While this mutation could be expected to have developed in isolated mutational events in most cases, we sought to determine the inheritance pattern of this mutation in a highly consanguineous population of Iran, in which inheritance of recurrent mutations causing recessive diseases is often due to transmission of a mutated haplotype from a common ancestor through generations, i.e.*,* the founder effect. The haplotype analysis of our three patients showed that the haplotypes harboring this mutation are identical by state, but not by descent, which supports the notion that this mutation is not the result of a founder effect in this Iranian cohort. Consequently, this mutation may occur sporadically in different populations, and therapeutic approaches targeting this mutation would likely have benefits in any population affected by HFS.

This study reports a novel causative mutation in HFS, contributes to our understanding of genotype-phenotype correlations for this syndrome, and improves our understanding of a recurrent *ANTXR2* gene mutation that may be a candidate for targeted therapy.

## Abbreviations

HFS: Hyaline fibromatosis syndrome
MAF: Minor allele frequencies
SNPs: Single nucleotide polymorphisms

## References

[1] Bell SE, Mavila A, Salazar R, Bayless KJ, Kanagala S, Maxwell SA (2001). Differential gene expression during capillary morphogenesis in 3D collagen matrices: regulated expression of genes involved in basement membrane matrix assembly, cell cycle progression, cellular differentiation and G-protein signaling. J Cell Sci.

[2] Scobie HM, Rainey GJ, Bradley KA, Young JA (2003). Human capillary morphogenesis protein 2 functions as an anthrax toxin receptor. Proc Natl Acad Sci U S A.

[3] Burgi J, Kunz B, Abrami L, Deuquet J, Piersigilli A, Scholl-Burgi S (2017). CMG2/ANTXR2 regulates extracellular collagen VI which accumulates in hyaline fibromatosis syndrome. Nat Commun.

[4] Tanaka K, Ebihara T, Kusubata M, Adachi E, Arai M, Kawaguchi N (2009). Abnormal collagen deposition in fibromas from patient with juvenile hyaline fibromatosis. J Dermatol Sci.

[5] Hanks S, Adams S, Douglas J, Arbour L, Atherton DJ, Balci S (2003). Mutations in the gene encoding capillary morphogenesis protein 2 cause juvenile hyaline fibromatosis and infantile systemic hyalinosis. Am J Hum Genet.

[6] Youssefian L, Vahidnezhad H, Aghighi Y, Ziaee V, Zeinali S, Abiri M (2017). Hyaline fibromatosis syndrome: a novel mutation and recurrent founder mutation in the CMG2/ANTXR2 gene. Acta Derm Venereol.

[7] El-Kamah GY, Fong K, El-Ruby M, Afifi HH, Clements SE, Lai-Cheong JE (2010). Spectrum of mutations in the ANTXR2 (CMG2) gene in infantile systemic hyalinosis and juvenile hyaline fibromatosis. Br J Dermatol.

[8] Vahidnezhad H, Ziaee V, Youssefian L, Li Q, Sotoudeh S, Uitto J (2015). Infantile systemic hyalinosis in an Iranian family with a mutation in the CMG2/ANTXR2 gene. Clin Exp Dermatol.

[9] Deuquet J, Lausch E, Superti-Furga A, van der Goot FG (2012). The dark sides of capillary morphogenesis gene 2. EMBO J.

[10] Yan SE, Lemmin T, Salvi S, Lausch E, Superti-Furga A, Rokicki D (2013). In-depth analysis of hyaline fibromatosis syndrome frameshift mutations at the same site reveal the necessity of personalized therapy. Hum Mutat.

[11] Deuquet J, Lausch E, Guex N, Abrami L, Salvi S, Lakkaraju A (2011). Hyaline fibromatosis syndrome inducing mutations in the ectodomain of anthrax toxin receptor 2 can be rescued by proteasome inhibitors. EMBO Mol Med.

